# Napping and Obesity in Adults – What do we Know?

**DOI:** 10.1007/s11892-024-01551-5

**Published:** 2024-08-15

**Authors:** Sasiwarang Goya Wannamethee

**Affiliations:** Department Primary Care and Population Health, University College London, Royal Free Campus, London, NW32PF UK

**Keywords:** Daytime Napping, Duration of Napping, Nighttime Sleep Duration, Obesity, Type 2 Diabetes

## Abstract

**Purpose of Review:**

To review the evidence on the relationship between daytime napping and obesity.

**Recent Findings:**

There is concern that napping may be harmful to metabolic health. Prospective studies have shown long time daytime napping (> 1 h) is associated with increased diabetes risk which may be partly associated with obesity. Evidence from numerous cross-sectional studies and meta-analyses of cross-sectional studies have shown that long time napping (> 1 h) but not short time napping is associated with increased risk of obesity, and this is seen worldwide. Inference regarding the nature of association from cross-sectional studies is limited; it is suggested the association is bidirectional. Prospective studies on the association between daytime napping and obesity are few and results unclear.

**Summary:**

Large longitudinal studies integrating daytime napping duration and night-time sleep behaviour and detailed information on lifestyle influences is needed to help elucidate further the associations of long time napping with obesity.

## Introduction

Daytime napping changes over the lifespan. Napping is common in infancy and early childhood, becomes less frequent in younger adults and becomes more common again in the elderly [1]. Daytime napping or siesta in adults is a common lifestyle practice in many countries worldwide and is thought to be a health promoting habit to improve mental health, cognitive performance, and work effectiveness particularly in sleep deprived populations such as night shift workers and airline pilots [1, 2]. However, this notion has been controversial because of the mounting evidence to suggest that long term daytime napping may be harmful to metabolic health and chronic disease risk [2, 3]. Numerous prospective studies and meta-analysis of prospective studies have shown daytime napping to be associated with increased Type 2 diabetes(T2DM) risk [4–10] and evidence indicate that long daytime naps (> 1 h/day) but not short naps are associated with increased T2DM risk [5, 6]. There have been suggestions that the association between napping and incident T2DM may be largely associated with obesity [7, 8, 10], a major causal risk factor for diabetes. Over the last decade much attention has turned to sleep patterns and obesity and a growing number of studies, mostly cross sectional in nature have investigated the association between napping and obesity particularly from studies in Chinese Populations. Numerous but not all studies have shown napping to be associated with obesity [11] and there is suggestion that duration of napping matters. Two recent meta-analysis on napping and obesity indicated that long daytime but not short daytime napping is associated with increased obesity risk [11, 12]. Napping is not a homogenous habit around the world. The effects of napping on obesity in mediterranean countries where siesta is a cultural habit or in China where midday napping is a common social habit may differ from effects in countries such as the UK and the US where siestas/napping are not culturally embedded. It is well documented that sleep loss or short sleep is associated with obesity and T2DM [13–15]; daytime napping may be a marker of insufficient sleep or poor sleep quality [16, 17]. It has also been suggested that the association between napping and obesity may be bidirectional with obesity leading to daytime napping [18]. In this article we review the evidence that daytime napping is associated with obesity in adults, whether the associations differ worldwide, whether duration of napping matters, and potential explanations which drive this association between napping and obesity.

### Daytime Napping and Obesity-Cross Sectional Studies

Evidence that napping is associated with obesity have come mainly from cross sectional studies. In 2022, a systematic review of 11 studies on napping and obesity conducted by Sun et al. as part of a comprehensive review and meta-analysis of studies on daytime napping and CVD risk factors and mortality, indicated inconsistent results between studies although most of the studies reviewed tend to show napping to be associated with higher odds of obesity [11]. In a meta-analysis of daytime napping and obesity conducted in 14 cross sectional studies including over 720,000 adults, the authors concluded that there was evidence that long daytime napping (> 1 h/d) is associated with obesity although there were inconsistencies between studies [11]. The association between daytime napping > 1 h and obesity were seen in all regions (Europe 5 studies, China 3 studies and Central Asia 1 study) and in both older and younger adults. However short daytime napping of < 1 h/d was not associated with higher odds of obesity. In a subsequent 2023 systematic review and meta-analysis of 12 studies which focussed specifically on napping and obesity conducted in China (5 studies), USA (5 studies), Spain (1 study) and the UK (1 study) Cai et al. concluded that daytime napping of more than 1 h was associated with increased odds of being obese [12]. No clear association was seen with short naps. Many of the studies overlapped in the two systematic reviews. In most of the studies included in the reviews daytime napping was self-reported. Subsequent to these reviews The Guangzhou Biobank cohort study of nearly 30,000 participants aged 50 + reported daytime nappers to have higher waist circumference (but not BMI) than non-nappers [10]. No information was available on duration of napping.

Daily naps are also a common habit in many Middle Eastern countries but studies from the Middle East were underrepresented in these two meta-analyses. Several studies conducted recently in the middle East/Persian populations have also shown daytime napping to be associated with increased obesity. In a study of 1683 adults aged > 37 years who participated in the Isfahan Study cohort study (Iran) daytime nappers showed higher BMI than non-nappers after taking education physical activity and smoking into account, but this was only seen in short nighttime sleepers (< 6 h/night) [19]. However, duration of daytime sleep was not available, and it is possible that the nappers with short sleep were more likely to nap longer; daytime napping in those with longer sleep are likely to have shorter naps hence the lack of association between napping and BMI. In The MASHAD study including over 9000 adults living in Iran, those who reported daily napping showed higher odds of obesity than non-nappers after adjustment for a range of factors including physical activity and education [adjusted odds ratio 95% CI for obesity = 1.34 (1.20–1.51]. No information was available for duration of sleep [20]. In another cross-sectional study of 400 young and middle-aged subjects (mean age 32.8 years) conducted in Muscat (Oman) [21], those who reported long afternoon napping (> 1 h) showed increased risk of obesity even after adjustment for possible confounders including physical activity. In the FASA PERSIAN COHORT study of over 10,000 subjects aged 35–70 years BMI increased with increasing daytime sleep duration even after adjustment for potential confounders including physical activity [22].

Other studies not included in the meta-analyses or systematic reviews conducted in Spain [23, 24] the UK [25], Japan [26] and the 26 countries study [27] have confirmed the findings of increased adiposity in those who napped a long time. In the PREDIMED-PLUS study of over 6000 adults aged 55–75 years from Spain, daytime napping > 90 min was associated with increased BMI [23]. In the ONTIME Study (Obesity, nutrigenetics, timing and Mediterranean Study) which included a mediterranean population of 3275 adults living in Spain (35% took siesta; 16% long siesta), long siesta (> 30 min) but not short siesta was associated with higher BMI levels compared to those reporting no siesta [24]. In a study of older British men aged > 71 years, napping > 1 h was associated with increased prevalence of obesity [25]. In a study of 189 community dwelling Japanese adults aged 80 years or older napping > 1 h showed increased odds of being obese compared to those reporting < 60 min nap even after adjustment for disease and physical activity [26]. In the 26-country study [27] comprising over 136,000 participants, the odds (95%CI) of being obese increased with duration of napping from 1.15 (1.11,1.20) in those napping < 1 h to 1.22 (1.15,1.13) in those napping > 1 h compared with non-nappers after adjusting for potential confounders.

Most all the studies to date on napping and obesity have relied on self-reporting. which might lead to recall bias. Reporting of daytime napping may be be influenced by sleep quality and emotion, potentially leading to obesity [12]. However, two studies from Japan and the US [26, 34] using objective measures of daytime napping (wrist actigraphy) have confirmed odds of obesity to increase with increase with increasing duration of daytime napping. Future studies incorporating objective measures, such as wrist actigraphy or polysomnography are needed which would provide more accurate and reliable information, to reduce potential bias associated with self-reported data.

### Confounders

Although many of these cross-sectional studies have adjusted for factors which may be associated with napping and obesity such as physical activity most studies have not taken other factors which may influence napping and obesity into account in particular diet and medical conditions. The ONTIME study showed that higher energy intake at lunch preceding the siesta and delayed meals at night played a mediating role in the napping obesity association [24]. Many conditions such as diabetes, obstructive sleep apnea, depression or anxiety disorder, dementia have an increased likelihood of napping, and these conditions are associated with obesity [28, 29].

### Effects of Sleep Loss and Daytime Napping on Obesity

Sleep is important in the restorative process of the body and its energy metabolism. Short nocturnal sleep is a common cause of daytime sleepiness, and it is becoming well recognised that sleep loss or short sleep duration is associated with obesity. A large body of epidemiologic evidence has linked short sleep duration with the development of obesity and other metabolic disorders such as diabetes [30–33]. Excessive daytime sleepiness is commonly assumed to be the result of disturbed or inadequate sleep; thus, napping may be a marker of sleep loss. However, several studies have examined the association between napping and obesity and the association is seen even after taking nighttime sleep, sleep apnoea or sleep factors that indicate insufficient nighttime sleep into account [20, 21, 27, 34–36] in multivariate analysis.

### Day Time and Nighttime Sleeping Patterns

Naps taken to compensate for poor nighttime sleep may differ from naps taken for enjoyment. Devine et al. have shown three common patterns of sleep behaviour infrequent nappers with good nighttime sleep, frequent nappers with good nighttime sleep and nappers with poor night-time sleep [37]. Only one cross sectional study to date has explored the interaction between nighttime sleep and daytime sleep on obesity. The influence of napping on overweight/obesity was seen in both those who reported short sleep (< 6 h) as well as those reporting 6–8 h’ sleep but not in long nighttime sleepers after adjustment [adjusted OR = 1.61 (1.07,2.41), 1.62 (0.93,2.82) and 1.33 (0.77,2.29)] for the three sleep groups respectively [19] although the findings in the 6–8 h’ sleep group was not statistically significant despite similar magnitude of association seen in the short sleep group. This may be due to the small number of men who report short sleep in the 6–8-h group. No detail was available on duration of daytime napping. It is possible that the association between duration of daytime napping and obesity may vary according to nighttime sleep patterns.

### Daytime Napping and Obesity—Prospective Studies

Cross-sectional studies cannot answer the question about the direction of the association between napping and obesity, but longitudinal studies may shed some light on the nature of the association. However, longitudinal studies on daytime napping and weight gain are few. Table 1 shows details of the longitudinal studies that have examined daytime napping and incident obesity or weight change. Several studies have shown long time napping to be associated with increased risk of developing the metabolic syndrome characterised by a set of cardiometabolic risk factors including abdominal obesity, hypertension, hypertriglyceridemia, and hyperglycaemia. In the Chinese Longitudinal study of over 13000 Chinese adults aged ≥ 45 years longer daytime napping (> 90 min/day) was significantly associated with higher incidence of metabolic syndrome (after 4 years follow-up). When examined by components longer daytime napping was associated with a 20% increased risk of central obesity [18]. In the Daily24 Multisite Cohort Study of 1016 adults from the US (median age 52 years) those who did not nap or napped less than 60 min had higher odds of weight loss (vs weight gain) compared to participants reporting longer nap durations [38]. The authors indicated that it is possible that longer daytime sleepiness causes weight gain through reduced physical activity. By contrast The Heinz Nixdorf Study in Germany of over 2800 adults aged 45–74 years showed no association between regular daytime napping and subsequent 5-year weight gain [39]. However, information on duration of napping was not available. This study compared all nappers to non-nappers without differentiating short nappers from long nappers. It is possible that short and long napping may have differing effects on weight gain; thus, combining the two groups may yield null associations. In the Spanish Sun Mediterranean Cohort of over 9400 adults aged 20–90 years living in Spain, long time nappers > 1 h/d showed no difference in odds of developing obesity compared to non-nappers but those who took short 30 min daily siesta had reduced odds of incident obesity (OR = 0.67,95%CI 0.46–0.96) [40]. However, when the combined effect of nighttime sleep and taking a siesta was assessed, those who reported short sleep (< 7 h/) and took more than 30 min daily siesta showed higher risk of becoming obese compared to those who slept > 7 h and taking a 30 min siesta; risk being over two-fold.

**Table 1:** Longitudinal studies on daytime napping and weight change and incident obesity

Study	N	Country	Follow-up	Outcome	Findings
China Health and Retirement Longitudinal Study (Wang et al. 2022) [18]	13821 Chinese adults aged ≥ 45 years	China	4 years	Incident central obesity	Extended nappers (> 90 min/d) vs non-nappers Adjusted relative risk 1.20 (1.038–1.397)
Daily24 Multisite Cohort Study (Hawkins et al. 2023) [38]	1016 adults median age 52 years	USA	5 years	Weight change over time Weight loss vs weight gain	Napping < 60 m/d was associated with higher odds of weight loss vs weight gain compared to napping > 60 m/d Adjusted odds ratio 1.49 (1.01,2.20)
Heinz Nixdorf Recall Study (Kowall et al. 2016) [39]	2837 participants aged 45–74 years	Germany	Median 5.1 years	Weight change between baseline and first follow-up visit	No association between regular day time napping and weight gain No information on duration of napping
The SUN Mediterranean Cohort (Sayon-Orea et al. 2013) [40]	9470 participants aged 20–90 years without obesity	Spain	Median 6.5 years	Incident cases of obesity	Compared to those with no siesta the HR (95%CI of becoming obese were Siesta < 30 min/d HR = 0.67,0.46–0.96 Siesta > 30- < 1 h/day HR = 0.93 (0.74–1.18) Siesta > 1 h/duration HR = 0.78 (0.51–1.19)

### Obesity as a Risk Factor for Daytime Sleepiness

Most of the evidence on napping and obesity come from cross sectional studies and reverse causation cannot be excluded as a possible explanation. It has been suggested that the association between napping and obesity may be bidirectional [18]. Obesity has been associated with an increase in sleep pressure and napping may be a consequence of increased adiposity. It is well recognised that obesity is a major risk factor for obstructive sleep apnea (OSA) which is considered an important contributor to sleepiness in obesity [41, 42]. Over 70% of people with OAS are overweight or obese; OSA often results in excess daytime sleepiness [41]. Earlier cross-sectional studies have shown excessive daytime sleepiness to be associated with obesity in the absence of sleep disordered breathing or sleep apnea [43, 44]. Several longitudinal studies have shown obesity or weight gain to be associated with excessive day time sleepiness. In the Sleep and HEalth in women ("SHE") cohort study of 7051 women (mean age 45.7 years) from Uppsala Sweden, obesity predicted incident excessive day time sleepiness [45]. In the Penn State Adult longitudinal cohort of ~ 1400 individuals aged > 20 years, obesity and weight gain were associated with the incidence of excessive day time sleepiness [46]. In the Sleep Health Heart Study of over 1400 participants aged 40–64 years and with no history of OSA 5-year weight gain was associated with worsening of daytime sleepiness [47]. A systematic review of weight loss interventions suggested that weight loss interventions improve daytime sleepiness supporting the causal effect of obesity on daytime sleepiness [48]. It is suggested that obesity alone can be a significant factor leading to daytime sleepiness because of circadian abnormality rather than just being secondary to nighttime sleep disturbance or OSA [43, 49].

### Short Naps and Obesity

There is no evidence that short naps usually defined as < 1 h/d is associated with obesity. By contrast short naps have been associated with reduced rate of metabolic diseases and lower weight gain [40, 50], and may be associated with other benefits (enhancing cognitive performance, reduce sleepiness and improving mood) [1]. Recent studies have shown short naps usually defined as < 30 min to have health benefits on cognitive and structural brain outcomes [51, 52].

### Genetic Studies of Excessive Daytime Napping with Obesity

Evidence that daytime napping may be causally related to obesity have come from genetic studies. Mendelian randomisation (MR) analysis has provided a cost efficient and robust approach to demonstrate causal links between napping and obesity. A recent large -scale study of napping habits and genomes from 452,633 UK Biobank participants identified the genetic variants influencing the likelihood to nap and suggest that daytime napping is partly regulated by genes and not just by environmental or behavioural choice and traits like laziness [53]. Genome wide association studies using mendelian randomisation analysis has suggested that habitual napping can be a causal factor for obesity [53–56] and for the metabolic syndrome characterised by central obesity, hypertension, or elevated triglycerides [57]. Genetic variants for daytime sleepiness also overlapped those for other diseases and lifestyle traits, with evidence that higher BMI and possibly diabetes are causally associated with increased daytime sleepiness [55–57] supporting a bidirectional relationship between daytime napping and obesity. However, the validity of an MR study for making causal inference assumes that the genetic variants have no other influence on the outcome, except through napping and that there are no confounders of the genetic variants-outcome association [58].

### Mechanisms

The exact mechanisms underlying the association between daytime napping and obesity are still not clear. Some possible biological mechanisms have been proposed for the association of daytime napping and obesity [12]. Recent studies suggest that long daytime napping may weaken the circadian rhythm and evidence form human and animal studies have suggested circadian mechanisms to be involved in the development of obesity [59–61]. However, the underlying biological basis remains complex. It has also been shown that excessive daytime napping is associated with elevated nighttime cortisol levels, which could increase insulin resistance and lead to abnormal blood lipids and fat distribution [62]. Long daytime napping may also increase sympathetic nervous system activity increasing cortisol levels which may promote eating behaviour and fat deposition leading to weight gain [63, 64]. In addition, long day time napping may reduce the total amount of calorie consumption and physical activity, which may cause decreased energy expenditure, leading to obesity [65].

### Conclusion and Future Direction

Overall, the findings to date suggest that long term daytime napping (> 1 h) but not short daytime naps is associated with obesity and the association maybe bidirectional. The association of daytime napping and increased risk of obesity in seen in different populations worldwide. There is no evidence that short naps(< 30 min/d) are associated with obesity and may even be beneficial in promoting cognitive and memory performance. Prospective studies on napping and incident obesity cases or weight gain are relatively few although genetic studies suggest a causal relationship between long time napping and obesity. Moreover, most studies have relied on self-reporting which may be subject to recall bias. Large longitudinal studies incorporating objective measures of day time napping are required to assess the influence of napping on incident obesity or weight gain considering behavioural factors and medical conditions that may influence the association between long time napping and obesity. Future prospective studies in different populations worldwide integrating daytime napping duration and night-time sleep behaviour is needed to help elucidate further the associations of napping with obesity. Experimental studies on napping and obesity are still lacking and the mechanisms underlying the association between long daytime napping and obesity remain unclear. Further clinical and experimental studies are warranted to provide insight as to how long naps may contribute to obesity.

## Data Availability

No datasets were generated or analysed during the current study.
